# Climate Driven Life Histories: The Case of the Mediterranean Storm Petrel

**DOI:** 10.1371/journal.pone.0094526

**Published:** 2014-04-11

**Authors:** Cecilia Soldatini, Yuri Vladimir Albores-Barajas, Bruno Massa, Olivier Gimenez

**Affiliations:** 1 Grupo de Ecología y Conservación de Islas, A.C., Ensenada, Baja California, México; 2 Department of Agricultural and Forest Sciences, University of Palermo, Palermo, Italy; 3 CEFE, UMR5175, Campus CNRS, Montpellier, France; Norwegian Polar Institute, Norway

## Abstract

Seabirds are affected by changes in the marine ecosystem. The influence of climatic factors on marine food webs can be reflected in long-term seabird population changes. We modelled the survival and recruitment of the Mediterranean storm petrel (*Hydrobates pelagicus melitensis*) using a 21-year mark-recapture dataset involving almost 5000 birds. We demonstrated a strong influence of prebreeding climatic conditions on recruitment age and of rainfall and breeding period conditions on juvenile survival. The results suggest that the juvenile survival rate of the Mediterranean subspecies may not be negatively affected by the predicted features of climate change, i.e., warmer summers and lower rainfall. Based on considerations of winter conditions in different parts of the Mediterranean, we were able to draw inferences about the wintering areas of the species for the first time.

## Introduction

In the face of the overwhelming data on the effects of climate change, it is crucial to understand how wildlife will react to such changes. Seabirds are considered sentinels of environmental change [1], as they range over large areas of the ocean and are therefore directly affected by any changes that may occur [1], [2], [3], [4]. Climate change affects oceanographic conditions and the availability of seabirds' prey, ultimately influencing population dynamics [5], [6], [7], [8]. Changes in fluid circulation may also induce positive consequences as it has been evidenced in the foraging efficiency and breeding success of albatrosses due to changes in wind regimes [9].

In view of the effects of environmental variability on the prey of seabirds, the climate-induced warming of oceanic surface waters to levels that exceed the boundaries of a fish species' thermal niche can have a direct effect on the distribution of the species. Even within this thermal niche, subtle (<0.5°C) temperature changes can have profound effects on growth, survival and reproduction [1], [10]. As a consequence, climate change can have an indirect impact on seabirds [11] influencing prey quality in marine food webs [12], [13] and complex bottom-up and top-down processes in ecosystems [14]. Climate factors may affect breeders directly through changing the distribution of important prey species or indirectly through affecting spawning and recruitment of important fish prey species giving lagged effects on seabird's demographic traits. Changes in prey abundance and quality during pre-breeding and chick rearing period may result in variation in adult body condition affecting their quality as breeders and or their survival. In that case changes that result in reduced caloric value of prey, compared to those occurring under “normal” conditions, may affect reproduction and chick survival [15], [16]. Chick survival may be indirectly affected by the same factors, influencing growth rate and body condition at fledging, but also by colony conditions in terms of humidity influencing thermo-regulation.

Long chick-rearing periods involving both parents are a characteristic of seabirds [17]. Reproductive output may therefore be strongly impacted by adverse conditions that force the birds to fast for longer periods or to make excessive metabolic effort [18], as a consequence juvenile survival and the overall population trend are at risk.

Cyclic and extreme climatic events are inherent to oceanic systems and have occurred long before human-induced global warming. Seabirds have evolved in this fluctuating environment and are negatively affected by the rapid increase of environmental stochasticity as a consequence of global warming [19].

In the Mediterranean, models have predicted an increase in temperature and a decrease in rainfall [20], which could produce the effects mentioned above, affecting the whole food web. In this context, we aimed at linking the effect of climatic conditions to the demography of seabirds with particular reference to the Mediterranean storm petrel (*Hydrobates pelagicus melitensis*). Our aims were: 1. to determine age of recruitment in Mediterranean storm petrel, 2. to investigate effects of environmental conditions in different time of the year on important demographic traits and 3. to reveal important wintering-area for this population. The last point is particularly challenging in species whose distribution is unknown and where technology is not yet available to track small animals at sea beyond the breeding period.

The Mediterranean storm petrel is a taxon distinguished both morphologically and genetically from the Atlantic populations [21], [22]. On the IUCN Red List the storm petrel is not considered to be under any threat and is thus categorized as Least Concern [23], but if we focus only on the Mediterranean subspecies, it may be endangered mainly due to habitat degradation and introduction of predators, such as rats and cats, requiring conservation awareness [24]. Breeding distribution and abundance is probably under estimated, due to the highly pelagic attitude and nocturnal behaviour [25]. The known Mediterranean population is estimated at between 11 000–16 000 breeding pairs, with three main population cores identified in Malta, Sicily and the Balearic Islands [26]. The current trend of the Mediterranean storm petrel populations is stable [26], although there is a lack of basin wide studies. In the few sites where mid-long term monitoring has taken place (Marettimo, Filfla and Benidorm), it has been observed that the colonies remain stable.

Dietary needs have evolved according to Mediterranean basin food-web and are mainly supplied by small pelagic fish [27]. Considering fluctuations of small pelagic fish whose biomass estimates resulted to be negatively correlated with the mean sea surface temperature [28], [29], [30], [31] we predict that climatic variation both in sea surface temperature and in environmental temperature and rainfall may affect storm petrel survival at different stages of their life cycle. Our hypothesis is that conditions experienced at fledging can influence first year survival and breeders may be affected by environmental conditions during the pre-breeding and breeding period with effects on age of recruitment and/or adult survival. We used temperature and rainfall in the pre-breeding and breeding period and sea surface temperature during winter in testing whether and in what measure birds are affected directly, chlorophyll concentration was used as indirect covariate.

We investigated the effects of climatic conditions on the demography of this taxon by analysing the life histories of almost 5000 individuals monitored since 1991 in the Egadi Archipelago, Italy. We then inferred that local conditions from the different areas during the winter can affect vital rates and analysed correlations between trends in different Mediterranean upwelling zones and storm petrel demography in order to predict the wintering areas.

## Materials and Methods

### Study area and species

The storm petrel (*Hydrobates pelagicus*) is the smallest procellariiform present in the Mediterranean Sea, where the subspecies *melitensis* lives. This species lays one egg in May-June and chicks fledge in late August-September. Mark-recapture data were obtained from Marettimo Is. (37°58′N, 12°03′E, Figure S1), Italy, between 1991 and 2012. The main colony, placed in a large cave accessible only from the sea, is the only accessible colony consisting of approximately 2500 pairs [25]. Recruitment in Marettimo is observed earlier than in other colonies [32], [33]. Each year, the colony was visited at least once between June and August, and adults and chicks were captured at nest and banded with metallic inox rings for a total of 4926 marked individuals. Sexing of the birds began in 2007 on chicks and adults following the methods already described in Albores et al. 2010 [34]. Adults caught at the colony are all breeders, as pre-breeders and skippers are not present.

### Climatic covariates

We used two types of covariates: time-dependent individual covariates and environmental covariates (Table 1). Individually based covariates follow individuals life history with no relation with other individuals while values of environmental covariates are shared by all individuals with no reference to their life history. In the case of time-dependent individual covariates, we had one value per individual, referring to the environmental conditions experienced at birth and the subsequent covariate values will characterize individual life history: the sequence of covariate values for each individual starts in the year of birth, hence birds born in different years have different sequences for the same covariate (e.g.: WNAO for the first 5 years of life of a bird born in 1995 will be: 1,363; −0,620; −0,067; −0,227; 0,643; while for a bird born in 1998 WNAO values will be: −0,227; 0,643; 1,303; 0,040; 0,237). We assumed these covariates to characterize individuals' life history (as for example in the case of individuals' weight), although they are not dependent on an individual's life history. In the case of environmental covariates, we had a single value per year that was shared by all individuals. Integrative indexes used as individual and external covariates were obtained performing Principal Components Analysis, PCA, see below for details on variables used [35].

**Table 1:** All covariates used in the analyses, and their main characteristics.

Kind of covariate	Name	Characteristics	label	Reference period	Available years	demographic parameters that could be influenced
Individual	Winter North Atlantic oscillation	global-scale covariates	WNAO	December-February	1991–2012	Survival
Individual	Temperature anomaly. Pre-Breeding period	local scale breeding ground covariate	PBT	March-April	1991–2012	Survival and breeding
Individual	Rainfall anomaly. Pre-Breeding period	local scale breeding ground covariate	PBR	March-April	1991–2012	Survival and breeding
Individual	North Atlantic oscillation. Pre-Breeding period	global-scale covariates	PBNAO	March-April	1991–2012	Survival and breeding
Individual	Integrative climatic index 1 Pre-Breeding period	PCA derived integrative covariates	PC1PB	March-April	1991–2012	Survival and breeding
Individual	Integrative climatic index 2 Pre-Breeding period	PCA derived integrative covariates	PC2PB	March-April	1991–2012	Survival and breeding
Individual	Temperature anomaly. Breeding period	local scale breeding ground covariate	BT	May-August	1991–2012	Survival and breeding
Individual	Rainfall anomaly. Breeding period	local scale breeding ground covariate	BR	May-August	1991–2012	Survival and breeding
Individual	North Atlantic oscillation. Breeding period	global-scale covariates	BNAO	May-August	1991–2012	Survival and breeding
Individual	Integrative climatic index 1 Breeding region	PCA derived integrative covariates	PC1B	May-August	1991–2012	Survival and breeding
Individual	Integrative climatic index 2 Breeding region	PCA derived integrative covariates	PC2B	May-August	1991–2012	Survival and breeding
Individual	West Sicily Rainfall index	PCA derived integrative covariates	WSR	May-April	1991–2012	Survival and breeding
External	Sea surface temperature	PCA derived integrative covariates	SST	December - February	2001–2010	Survival
External	Chlorophille concentration	PCA derived integrative covariates	CHL	December - February	2001–2010	Survival

In the table we reported time-dependent individual covariates as “individual” and environmental covariates as “external”.

We defined four time-dependent individual covariates (WSR, western Sicily rainfall, WNAO, winter NAO, PB, pre-breeding period integrative covariate, and B, breeding period integrative covariate) to test climate effects on the recruitment probability and survival of chicks and adults.

- WNAO: we selected the December through February winter North Atlantic Oscillation [36] due to its broad geographic coverage and used the average value of the selected period.
- WSR: to define the integrative covariate a PCA was performed on rainfall data from the four meteorological stations closest to the island of Marettimo: Erice, Trapani, Marsala, and Mazara del Vallo (Figure S1) [37], [38]. The analysis considered the total rainfall recorded in 12 months relative to the life cycle year (May to April, considering the year starting with egg laying).
- PB and B: to incorporate the uncertainty with which climatic variables affect the pre-breeding (March-April) and breeding (May-August) periods, we included temperature, rainfall, and NAO anomalies in the PCAs to calculate the integrative covariates as Pre-Breeding, PB, and Breeding, B, indexes.

To test winter period climate effects on adult survival and identify potential wintering sites for the species, we considered two external covariates defined as environmental covariates: SST, sea surface temperature, and CHL, chlorophyll concentration. We considered time series from 2001 to 2010 of SST and CHL as proxies of environmental conditions [39] from five areas with upwelling conditions in different regions of the Mediterranean Sea (Alboran Sea, Balearic Sea, Sicilian Channel, Adriatic Sea and Aegean Sea) [40]. To test the effect of winter conditions, we analysed data from December to February, performing 2 PCAs for each sea area (1 PCA for sea surface temperature, SST, and 1 PCA for chlorophyll concentration, CHL, each one based on 5 points). We then used the PC factors as external covariates in the model for a total of 11 covariates tested.

### Survival and recruitment estimate from encounter history

We applied a multistate mark-recapture analysis to estimate chick and adult survival, to assess recruitment and to evaluate the potential effects of time and sex on these variables [41], [42]. Recruitment probability is given by the probability of transition from the non-breeder to the breeder state and can vary according to age and to time. We constrained variation with age by considering that recruitment probability stabilizes to a constant value from a given age onward. We defined first year survival after fledging as chicks survival and adults survival that of adults of subsequent age classes. The assumptions underlying our model are: a. detection probability of non-breeders and skippers is zero; b. once in the breeder state, individuals cannot go back to the non breeder state; c. there is an age from which the probability of becoming a breeder (probability of transition from non breeder to breeder) levels off at a constant value. As a consequence, at a given sampling occasion, an animal may be a pre-breeder (a juvenile of one year or more, not yet recruiting, state PB), breeder (adults after first breeding are generally considered breeders, state B), or dead (state dead, unobservable). The following observations may be made: ‘1’ (if detected as chick), ‘2’ (if detected as adult) and ‘0’ (if not detected). We tested the full recruitment age (the age at which any bird has become a breeder) and the age of full breeding (considering the probability of becoming a breeder as a function of age). To address age variation in the probability of becoming a breeder, we used only individuals of known age, i.e., only those ringed as chicks (3135), considering ringing as chicks as first capture event. We define the initial state vector Π, the transition matrix ΦΨ (survival multiplied by transition between states conditional on survival) and the event matrix *B*. 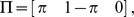


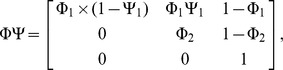


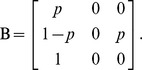



Columns of the matrix Π and 
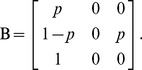
 correspond respectively to state PB, B and dead, columns of the matrix *B* correspond to the observations ‘non detected’ and ‘detected’ while rows of the matrices 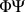 and *B* correspond respectively to state PB, B, and dead. 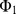
denotes the pre-breeder survival, 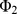 the adult survival, 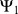
is the probability of becoming a breeder and *p* is the detection probability.

Individuals are first captured as PB and then become B; π denotes the proportion of newly marked in state PB. Φ_1_×Ψ_1_ represents the probability that a pre-breeder survives and attempts breeding. For an individual in state B, Φ_2_ represents the probability that a breeder survives. After the first capture, pre-breeders are not observed as they do not attend the colony; skippers are not present at the colony; we then estimated breeders detection as the detection parameter in the “breeder” state is the product of the probability of not skipping breeding and of the probability of detection given breeding.

Goodness-of-fit tests [43] were performed using U-CARE [44]. The results of the GOF test are summarised in Table 2.

**Table 2:** Results of goodness of fit test for the multistate model.

Test component	X^2^	d.f.	P
Global test	48.568	52	0.610
Test 3G (test for transience)	22.264	23	0.504
Test M (test for trap-dependence)	26.304	29	0.609

We analysed 25 model combinations in which Ψ was modeled as a constant or functions of age and time, either singly, additively, or with interactions. The model assumes that there are two age classes (juvenile and adult) and there is an age from which the probability to breed *a*i does not change. Full recruitment age is the age at which any individual has become a breeder, it is equivalent to age “m” as defined by Gauthier and colleagues [45], which considers age “m” as the age at which recruitment probability can be considered constant for all subsequent ages. Juvenile survival and breeder recapture probabilities were allowed to be constant or to vary with time.

We distinguished the first from the following encounter occasions, as the encounter history is conditional on being caught in the first period and the following detection probabilities depend on the state and the time occasions. We started running a model assuming that there is no change after 10 years. As we obtained that after age 7 there is no improvement in model fitting we reduced the full breeding age to 7 (namely the 6th year of age) (Table 3). For a full breeding probability we fixed Ψ relative to this hypothetical age 1. Thus we tested which is the full recruitment age (probability of becoming a breeder) by fixing to 1 the potential age of full recruitment, specifying initial parameter values we constrained beta values before running the model.

**Table 3:** Selection of the full breeding age (f.b.), only best models are presented.

Model	Deviance	NP	AIC	ΔAIC	AIC weight
**f. b. year 7 (age 6)**	**3127.74**	**29**	**3185.74**	0.00	1.000
f. b. year 5 (age 4)	3224.01	27	3278.01	92.27	0.000
f. b. year 6 (age 5)	3224.01	28	3280.01	94.27	0.000
f. b. year 8 (age 7)	3224.01	30	3284.01	98.27	0.000
f. b. year 9 (age 8)	3224.01	31	3286.01	100.27	0.000

Deviance (Deviance), number of parameters (NP) and AIC [47] are reported. Preferred model is in bold.

We tested the time effect on survival and the effect of time and sex (the last as group effect) on recruitment only. We limited the tests in this way because we primarily sexed adults and we have information on chicks sex only after 2007.

Bearing in mind previous results, to assess climatic effects on survival and recruitment, model selection began with the model with age and time effects on survival and sex effect on recruitment age. More in details, after testing the single effect of the individual covariates on survival (Φ) and recruitment (Ψ) we tested the additive effects of different individual covariates and time on survival (Φ) and the additive effects of different individual covariates, time and sex on recruitment (Ψ).

Models were fitted with the E-SURGE program [46] (for model implementation in E-Surge see Material S1). Model selection was based on the Akaike Information Criterion (AIC) [47], [48].

### Effects of winter climate on survival over time

Because data on environmental conditions are available only from 2001 to 2010, we used a subset of birds ringed as chicks and adults in the same period (2918), modelling potential effect of covariates only on adults survival. We carried out goodness-of-fit for the single state model using U-CARE [6]. Results of the GOF test are summarized in Table 4. Due to the presence of transient individuals, we used 2-age-class models and a variance inflation factor (c-hat = 1.4). Following the single state, as simplification of the scheme reported above, we implemented the model in program E-SURGE [44] (for model implementation in E-Surge see Material S1).

**Table 4:** Results of the GOF test for the single-state model.

Test component	X^2^	d.f.	P
Global test	233.82	61	0
Test 3.SR (test for transience)	162.40	10	0
Test 2.CT (test for trap-dependence)	9.23	9	0.42
Global test (corrected for transience)	71.42	51	0.03

We analysed 24 model combinations in which Φ was modeled as a constant or function of time, we then explored singly and additively interaction with environmental (external) covariates dependent models. The model assumes that there are two age classes (young, first year of age, and adult, older than 1 year), survival and breeder recapture probabilities were allowed to be constant or to vary with time.

Model selection procedure: model selection was based on the Akaike's information criterion (AIC) [47], adjusted for overdispersion (Q). After identifying time-dependent parameters, we investigated the effects of SST and CHL in describing the time variation with an analysis of deviance (ANODEV) and calculated the proportion of variance explained by the environmental (external) covariates using the R^2^ statistic [35].

### Ethics Statement

Field work was carried out under permission from the Marine Protected Area. Permits included obtaining DNA samples for sexing. There were no ethics committee approval requirements to carry out this project due to the minimum handling of the individuals that was carried out according to Italian and Sicilian region legislation (LN 157/92, LR 105/99 and LR 74/2012) by certified ringers authorized by the Institute for Research and Environmental Protection (ISPRA) to handle adults and chicks.

## Results

Individual covariates were defined and we made sure that the covariates used as integrative climatic indexes were not inter-correlated (Table 5). WSR is represented by the first principal component explaining 86,49% of the total variance (figure S2a). PB is represented by principal components 1 and 2 (PC1PB and PC2PB hereafter). The first factor, explaining 42.57% of the total variance, is correlated positively with temperature and negatively with rainfall, whereas factor 2, explaining 32.19% of the total variance, is positively correlated with the NAO (figure S2b). During the pre-breeding period, warm and dry conditions are associated with a high NAO, whereas cold and wet conditions are associated with a low NAO. B is represented by the first principal component (PC1B hereafter, explaining 45.97% of the total variance), which is positively correlated with rainfall and the NAO. B is also represented by the second principal component (PC2B hereafter, explaining 33.42% of the total variance; figure S2c), which is positively correlated with temperature. These results indicate that during the breeding period (i.e., the chick-rearing period), warm and dry conditions are associated with a low NAO, and cold and wet conditions are associated with a high NAO. Trends of individual and environmental covariates are summarised in figures S3a and S4a.

**Table 5:** Correlations between climatic covariates.

Pearson Correlation	Correlation pattern (Pearson's *r* below the diagonal; *P* value of a *t*-test for *H_0_*: *r* = 0 above the diagonal)												
Climatic covariate	Label	WNAO	PBTan	PBRainan	PBNAO	PC1PB	PC2PB	BTan	Brainan	BNAO	PC1B	PC2B	WSR
**Winter North Atlantic oscillation (December-February)**	**WNAO**		0.919	0.214	0.409	**0.293**	**0.585**	0.295	0.560	0.663	**0.855**	**0.496**	**0.026**
Temperature anomaly. Pre-Breeding period (March-April)	PBT	0.023		*0.282*	*0.814*	0.000	0.146	0.610	0.793	0.328	0.844	0.351	0.138
Rainfall anomaly. Pre-Breeding period (March-April)	PBR	−0.276	*−0.240*		*0.691*	0.000	0.483	0.482	0.968	0.889	0.760	0.590	0.000
North Atlantic oscillation. Pre-Breeding period (March-April)	PBNAO	0.185	*0.053*	*−0.090*		0.073	0.000	0.819	0.817	0.529	0.689	0.611	0.097
**Integrative climatic index 1 Pre-Breeding period (March-April)**	**PC1PB**	**0.235**	0.736	−0.764	0.389		**1.000**	0.956	0.955	0.401	**0.676**	**0.715**	**0.000**
**Integrative climatic index 2 Pre-Breeding period (March-April)**	**PC2PB**	**0.123**	−0.320	0.158	0.916	**0.000**		0.871	0.765	0.803	**0.792**	**0.792**	**0.599**
Temperature anomaly. Breeding period (May-August)	BT	−0.234	0.115	0.158	0.052	−0.013	0.037		*0.400*	*0.989*	0.059	0.000	0.535
Rainfall anomaly. Breeding period (May-August)	BR	−0.131	−0.059	−0.009	0.052	−0.013	0.068	−*0.189*		*0.133*	0.000	0.986	0.426
North Atlantic oscillation. Breeding period (May-August)	BNAO	0.098	0.219	−0.032	0.142	0.188	0.057	*0.003*	*0.330*		0.000	0.019	0.491
**Integrative climatic index 1 Breeding region (May-August)**	**PC1B**	**0.041**	0.045	−0.069	0.090	**0.094**	**0.060**	−0.409	0.832	0.721		**1.000**	**0.948**
**Integrative climatic index 2 Breeding region (May-August)**	**PC2B**	−**0.153**	0.209	0.122	0.115	**0.083**	**0.060**	0.869	−0.004	0.497	**0.000**		**0.847**
**West Sicily Rainfall index**	**WSR**	−**0.473**	−0.327	0.719	−0.363	−**0.729**	−**0.119**	0.140	0.179	−0.155	−**0.015**	**0.044**	

The values of sets of covariates describing climatic conditions in the breeding area during the pre-breeding period and the breeding period, respectively, are in italic. Bold type highlights the covariates retained for the analysis of climatic effects on survival and recruitment.

By fixing to 1 the potential age of becoming a breeder (full recruitment) or the age at which all the birds of the colony may be considered breeders, we found that the 2^nd^ year is the age at which most birds start to recruit with a small difference with the 3^rd^ year of age (AIC = 3176.14 and AIC weight  = 0.342; ΔAIC = 1.60 and AIC weight  = 0.154 for 3^rd^ year of age, but see Table 6 for more details) suggesting that at the 2^nd^ and, in smaller proportion, at the 3^rd^ year of age are Marettimo's storm petrels are becoming breeders. In table 6 both the results of full recruitment age identification and of sex effect on recruitment age are reported. The best model considered constant annual survival trends, whereas recruitment was affected by sex, with males recruiting earlier than females (see also figure S5). Recruitment probability in males is slightly higher than in females during the first 3 years of age (in average 4.8%), this difference is no more perceivable from the 4^th^ year. Full breeding age in the Marettimo colony is at the 6^th^ year of age (Table 3).

**Table 6:** Selection of the full recruitment (f.r.) age and test of time effect on survival and sex effect on recruitment age, only best models are presented.

Model	Deviance	NP	AIC	ΔAIC	AIC weight
**Φ_t_ Ψ_sex_**	**3002.59**	**52**	**3106.59**	**0.00**	**0.827**
Φ_t_ Ψ	3011.83	49	3109.83	3.24	0.164
Φ_t_ Ψ f.r. year fixed to 3	3019.74	48	3115.74	9.15	0.008
**f.r. year 3 (age 2)**	**3128.14**	**24**	**3176.14**	**0.00**	**0.342**
f.r. year 4 (age 3)	3127.74	25	3177.74	1.60	0.154
f.r. year 5 (age 4)	3127.74	26	3179.74	3.60	0.165
f.r. year 2 (age 1)	3134.77	23	3180.77	4.63	0.034

Deviance (Deviance), number of parameters (NP) and AIC [47] are reported. Preferred models are in bold.

We found high mortality in juveniles. The average first year survival after fledging was 0.222 (±0.093) and adults survival was 0.923 (±0.060). Survival was highly variable (figure S3b), especially for juveniles.

Considering single and additive effects of group (sex) and individual covariates (local climatic descriptive indexes) we found that recruitment differed between sexes with males recruiting earlier. Pre-breeding season conditions with colder springs was negatively affecting recruitment (Table 7), whereas survival in first year after fledging and adults was affected positively by warm breeding-period conditions and negatively by abundant rainfall explaining the correspondence of the drops in figure S3b with higher B and WSR and lower PB values in figure S3a.

**Table 7:** Model selection results, only best models are presented.

Model	Deviance	NP	AIC	ΔAIC	AIC weight
**Φ_B1+B2+t+WSR_ Ψ_PB1+PB2+sex_**	**2984.08**	**55**	**3094.08**	**0.00**	**0.842**
Φ_B1+B2+t_ **Ψ**_PB1+PB1+sex_	2989.76	54	3097.76	3.68	0.134
Φ_B1+B2+t_ **Ψ**_PB1+PB2_	2999.35	51	3101.35	7.27	0.022
Φ_t_ **Ψ**_sex_	3002.59	52	3106.59	12.51	0.001
Φ_t_ **Ψ_i_**	3011.83	49	3109.83	15.75	0.000
Φ_B1+B2_ **Ψ_i_**	3104.72	29	3162.72	68.64	0.000
Φ**_i_** **Ψ**_PB1+PB2_	3112.46	30	3172.46	78.38	0.000
Φ**_i_** **Ψ**_sex_	3112.68	32	3176.68	82.60	0.000
Φ_PB1+PB2_ **Ψ_i_**	3121.82	28	3177.82	83.74	0.000
Φ_WSR_ **Ψ_i_**	3124.11	27	3178.11	84.03	0.000
Φ_WNAO_ **Ψ_i_**	3126.47	27	3180.47	86.39	0.000
Φ**_i_** **Ψ_i_**	3127.74	29	3185.74	91.66	0.000

Climatic (PB: Pre-Breeding, B: Breeding, WSR: rainfall, WNAO: winter NAO), “time”, and constant effects “i” were considered on survival, Φ, *Ψskipping* the transition probability from state pre-breeder to breeder, Ψ (“+” denotes additivity). Deviance (Deviance), number of parameters (NP), ΔAIC and AIC weight are reported.

After defining environmental covariates as SST and CHL PCA factors (Table 8) we obtained cues on their effects on adults survival and encounter probability. We obtained that SST and CHL, are not correlated (Spearman R = −0.336, p = 0.321, figure S4a) giving strength to the models involving them both. We can confirm that SST and CHL may influence storm petrels ecology during winter time [39]. A higher sea surface temperature and lower chlorophyll concentration were associated with lower encounter rates during the subsequent breeding period (figure S4b) and a higher encounter rate after favourable years. Winter sea conditions in different areas of the Mediterranean affected the survival of the Marettimo birds. In particular, we found that periods of low productivity (high SST and low CHL) in the Alboran Sea negatively influenced adult survival (Table 9). In fact we found a significant effect of Alboran Sea conditions (AIC = 4175.04 and compared to time effect: ΔAIC = 1.37, AIC weight 0.335, F_2,15_ = 14.385, p = 0.004), with 62% of the temporal variance in adult survival explained by the Alboran sea-surface temperature and chlorophyll variation. When considering neighbouring models (SST and CHL covariates considered separately) we obtained that SST has a stronger effect (AIC = 4175.04 and compared to time effect: ΔAIC = 1.37, AIC weight 0.335, F_2,15_ = 14.385, p = 0.004, R^2^ = 62%) than CHL (AIC = 4183.76 and compared to time effect: ΔAIC = 10.09, AIC weight 0.001, F_2,15_ = 5.560, p = 0.040, R^2^ = 38%). Thus SST alone explains a large percentage of the total variation on adult survival. This finding suggested that the Alboran Sea was the most probable wintering site, and that environmental conditions there affect overwintering conditions in the Mediterranean storm petrel population of Marettimo.

**Table 8:** Percentage of variance expressed by each principal component factor of the PCAs performed (Alboran Sea, Balearic Sea, Sicilian Channel, Adriatic Sea, and Aegean Sea: Al, L, S, Ad, Ae, respectively; sea surface temperature: SST and chlorophyll: CHL).

ext cov	% cov
**LsstPC1**	70.069
**LchlPC1**	44.937
**LchlPC2**	29.623
**AlsstPC1**	82.34
**AlchlPC1**	86.231
**AdsstPC1**	87.756
**AdchlPC1**	51.557
**AdchlPC2**	29.163
**SsstPC1**	88.377
**SchlPC1**	78.176
**AesstPC1**	88.872
**AechlPC1**	92.015

**Table 9:** Model selection results.

Model	Deviance	NP	AIC	ΔAIC	AIC weight
**time eff**	4125.67	24	4173.67	0.00	0.664
**Al sst chl**	**4145.01**	**15**	**4175.04**	**1.37**	**0.335**
**Al sst**	4145.01	15	4175.04	1.37	0.335
**Al chl**	4156.74	15	4183.76	10.09	0.001
**L sst chl**	4155.86	15	4185.86	12.19	0.001
**Ad sst chl**	4161.70	15	4191.70	18.03	0.000
**Ae sst chl**	4167.40	15	4197.40	23.73	0.000
**S sst chl**	4170.41	15	4200.41	26.74	0.000
**Constant**	4175.93	14	4203.93	30.26	0.000

Environmental, “time”, and constant effects “i” were considered on survival, Φ, with climatic covariates in 5 areas of the Mediterranean (Al: Alboran, L: Balearic, Ad: Adriatic, Ae: Aegean, S: Sicilian Sea). Deviance (Deviance), number of parameters (NP), ΔAIC and AIC weight are reported.

## Discussion

### Recruitment age determination

Petrels are long-lived organisms with delayed sexual maturity. A prolonged pre-recruitment period and extended lifespan provide seabirds with sufficient time to explore marine habitats and to gather crucial information about prey patches and the spatio-temporal variability in their availability [5]. Although we found survival values similar to those reported by a previous study [32], the recruitment age for the Marettimo colony was lower, perhaps due to the greater number of individuals located in this colony and the much greater intra-individual variation that resulted. Marettimo colony study conditions and structure are different from most of known colonies, being the colony placed in a big and completely dark cave accessible only from the sea (thus predator-free), and with a large number of accessible nests. The colony characteristics make it very different from other monitored Mediterranean colonies such as Filfla, Malta, where nests are scattered in single inaccessible burrows in the rocks [24], and Benidorm, Spain, where the colony is small (ca 300 nests) and in two caves easily accessible for predators [49]. The database we used in this study is based on a more complete and longer time series (21 years with one missing year vs 15 years with two missing years) than that used previously for the same colony in a comparative study [32]. This allowed us to investigate and describe cues of the subspecies life history, thanks to predators absence, making it possible to infer about chicks natural mortality, and the long time series allowing correlational analyses with climatic conditions trends. The population studied showed that the pre-recruitment period may be shortened by unfavourable environmental and climatic conditions that may have lead to earlier maturation as a consequence of lower adult survival (figure S3b). Long-lived birds invest more in survival than fertility [50]. However, this study found that storm petrel tend to recruit earlier in this colony compared to other monitored colonies throughout the Mediterranean [32]. It is possible that this result is due to the effects of environmental conditions on survival.

### Effects of environmental conditions on demographic traits

Survival exhibited two troughs (figure S3b), which might be due to factors related to colony dynamics (internal) and to environmental factors (external). The influence of climate on demography is represented by the finding that the colony region's climatic conditions affect the recruitment age and survival of chicks and adults. In particular, chick survival was affected by breeding-period conditions and rainfall. Abundant rainfall before and cold weather during the chick-rearing period resulted in higher chick mortality, as observed in 2010. These conditions may have produced wet conditions in the colony caves and reduced thermoregulation capabilities, inducing higher metabolic costs for chicks and parents [18]. In fact, in 2010, a year with higher rainfall and lower temperature, we observed exceptionally high chick mortality in the field (only 20 fledged in the surveyed area of the colony), while we usually don't find dead chicks when visiting the colony. In the view of our results warmer summers and lower rainfall predicted as a consequence of the changing climate [51] will not have a negative effect on juvenile survival rate of the Mediterranean subspecies.

Adult survival was directly affected by the climatic conditions at the time of breeding due to the adult's needs to meet its own energetic requirements as well as those of the chick. Males tend to recruit earlier than females and recruitment was reduced in years with adverse prebreeding conditions, suggesting that younger birds decided to delay recruitment to wait for better condition or more productive years. Carryover effects for the winter might occur as a consequence [52]. It is noteworthy the fact that males of Mediterranean storm petrels are smaller than females [53] and consequently adults may rear the chicks with fewer energy expenditure.

### Identification of wintering-area

The original aspect of our study is the use of statistical models to identify seabird wintering areas, as illustrated for the storm petrel, a species difficult to follow at sea due to lack of technology, as geolocators or satellite tracking devices are not small enough to be used on this species. The trends of sea surface temperature and chlorophyll [39] (figure S4a) may influence storm petrel survival. In particular, the conditions in the Alboran Sea zone resulted to be influencing adult survival. Considering sea surface temperature and chlorophyll concentration effects separately, we observed that SST has a stronger direct effect while the CHL direct effect is weaker (Table 9). It is still unknown what the Mediterranean storm petrel feeds on during winter. Our results suggest that they probably feed mainly on small fishes, not directly affected by CHL, as during the breeding period [27], and not on zooplankton that would be directly affected by CHL concentration. Sea conditions may induce birds to skip reproduction in years with high surface temperatures and low chlorophyll levels at wintering sites, resulting in lower encounter rates during the subsequent breeding season. This would explain the drops in encounter rate observed in breeding seasons after high sea surface temperature and low chlorophyll concentration in winter conditions (figure S4a and b). Our results highlighted the relationship between environmental covariates and encounter rates, as also found in the Pacific and other studies on impacts of climate change on avian populations [11], [54].

Finally with respect to predicted features of climate change [51], as we observed that colony region conditions affect chick and adult survival, whereas conditions in the wintering areas affected adult survival, we can conclude that the stability of the Alboran sea surface temperature due to anticyclonic systems [55] may result in continuous upwelling in the area thus resulting in stability of survival of adult storm petrels during winter while the anticipated decrease in precipitation and warmer summers [20] will positively affect chick survival. Although we did not consider climatic effects on the food chain our result suggest future demographic stability and an improvement of the species status. Further research on climate change effects on the food chain and consequent effects on the Mediterranean storm petrel are currently missing and would help to forecast the species future conditions.

## Supporting Information

Figure S1 **Colony and meteorological stations location.**
(TIF)

Figure S2 **Correlation graphs for principal component analysis (PCA) for building integrative climatic indices.** a) PCA of West Sicily rainfall data; b) PCA of the pre-breeding climatic variables (temperature, rainfall, and NAO); c) PCA of the breeding season climatic variables (temperature, rainfall, and NAO). Abbreviations are as in Table 6.
(TIF)

Figure S3 **Trends of individually based covariates and demographic traits.** a) Time series of climatic indices considered as candidate covariates in the analysis of survival and recruitment (PB: Pre-Breeding, B: Breeding, WSR: rainfall, WNAO: winter NAO). b). Estimated survival at Marettimo, confidence intervals in dotted lines.
(TIF)

Figure S4 **Trends of environmental covariates and demographic traits.** a) Time series of sea surface temperature, SST, and chlorophyll concentration, CHL, in the Alboran Sea. b) Estimated survival and encounter rate at Marettimo's colony using Alboran Sea climatic conditions (CI values are nil thus not visible in the graph).
(TIF)

Figure S5 **Recruitment probability of males and females storm petrels.**
(TIF)

Material S1 **Detailed descriptions of model implementation in E-Surge.**
(DOCX)

## References

[1] Barbraud C, Rolland V, Jenouvrier S, Nevoux M, Delord K, et al (2012) Effects of climate change and fisheries bycatch on Southern Ocean seabirds: a review. Marine Ecology-Progress Series 454: 285–307.

[2] Wolf SG, Sydeman WJ, Hipfner JM, Abraham CL, Tershy BR, et al (2009) Range-wide reproductive consequences of ocean climate variability for the seabird Cassin's Auklet. Ecology 90: 742–753. 19341144 10.1890/07-1267.1

[3] Thompson DR, Furness RW, Monteiro LR (1998) Seabirds as biomonitors of mercury inputs to epipelagic and mesopelagic marine food chains. Science of the Total Environment 213: 299–305.

[4] Watanuki Y, Ito M (2012) Climatic effects on breeding seabirds of the northern Japan Sea. Marine Ecology-Progress Series 454: 183–196.

[5] Daunt F, Afanasyev V, Adam A, Croxall JP, Wanless S (2007) From cradle to early grave: juvenile mortality in European shags Phalacrocorax aristotelis results from inadequate development of foraging proficiency. Biology Letters 3: 371–374. 17504733 10.1098/rsbl.2007.0157 PMC2390668

[6] Frederiksen M, Harris MP, Daunt F, Rothery P, Wanless S (2004) Scale-dependent climate signals drive breeding phenology of three seabird species. Global Change Biology 10: 1214–1221.

[7] Frederiksen M, Daunt F, Harris MP, Wanless S (2008) The demographic impact of extreme events: stochastic weather drives survival and population dynamics in a long-lived seabird. Journal of Animal Ecology 77: 1020–1029. 18557956 10.1111/j.1365-2656.2008.01422.x

[8] Furness RW, Tasker ML (2000) Seabird-fishery interactions: quantifying the sensitivity of seabirds to reductions in sandeel abundance, and identification of key areas for sensitive seabirds in the North Sea. Marine Ecology Progress Series 202: 253–264.

[9] Weimerskirch H, Louzao M, de Grissac S, Delord K (2012) Changes in Wind Pattern Alter Albatross Distribution and Life-History Traits. Science 335: 211–214. 22246774 10.1126/science.1210270

[10] Gremillet D, Boulinier T (2009) Spatial ecology and conservation of seabirds facing global climate change: a review. Marine Ecology-Progress Series 391: 121–137.

[11] Jenouvrier S (2013) Impacts of climate change on avian populations. Global Change Biology 19: 2036–2057. 23505016 10.1111/gcb.12195

[12] Frederiksen M, Edwards M, Richardson AJ, Halliday NC, Wanless S (2006) From plankton to top predators: bottom-up control of a marine food web across four trophic levels. Journal of Animal Ecology 75: 1259–1268. 17032358 10.1111/j.1365-2656.2006.01148.x

[13] Rossoll D, Bermudez R, Hauss H, Schulz KG, Riebesell U, et al. (2012) Ocean Acidification-Induced Food Quality Deterioration Constrains Trophic Transfer. Plos One 7.. 10.1371/journal.pone.0034737 PMC3324536 22509351

[14] Frederiksen M, Furness RW, Wanless S (2007) Regional variation in the role of bottom-up and top-down processes in controlling sandeel abundance in the North Sea. Marine Ecology Progress Series 337: 279–286.

[15] Wanless S, Harris MP, Redman P, Speakman JR (2005) Low energy values of fish as a probable cause of a major seabird breeding failure in the North Sea. Marine Ecology-Progress Series 294: 1–8.

[16] Blight LK (2011) Egg Production in a Coastal Seabird, the Glaucous-Winged Gull (Larus glaucescens), Declines during the Last Century. Plos One 6.. 10.1371/journal.pone.0022027 PMC3138773 21789207

[17] Schreiber EA, J B, editors (2002) Biology of Marine Birds. Boca Raton: CRC Press.

[18] Weimerskirch H, Cherel Y, CuenotChaillet F, Ridoux V (1997) Alternative foraging strategies and resource allocation by male and female Wandering Albatrosses. Ecology 78: 2051–2063.

[19] Alley RB, Marotzke J, Nordhaus WD, Overpeck JT, Peteet DM, et al (2003) Abrupt climate change. Science 299: 2005–2010. 12663908 10.1126/science.1081056

[20] Blade I, Liebmann B, Fortuny D, van Oldenborgh GJ (2012) Observed and simulated impacts of the summer NAO in Europe: implications for projected drying in the Mediterranean region. Climate Dynamics 39: 709–727.

[21] Cagnon C, Lauga B, Hemery G, Mouches C (2004) Phylogeographic differentiation of storm petrels (Hydrobates pelagicus) based on cytochrome b mitochondrial DNA variation. Marine Biology 145: 1257–1264.

[22] Lalanne Y, Hemery G, Cagnon C, D'amico F, D'Elbee J, et al (2001) Discrimination morphologique des sous espèces d'Oceanite tempete: nouveaux résultats pour deux populations méditerranéennes. Alauda 69: 475–482.

[23] IUCNRed List of Threatened Species Available: www.iucnredlist.org, Accessed on 1 Oct 2014

[24] Massa B, Sultana J (1991) Status and conservation of the Storm Petrel Hydrobates pelagicus in the Mediterranean. Il Merill 27: 5.

[25] Albores-Barajas YV, Massa B, LoCascio P, Soldatini C (2012) Night surveys and smell, a mixed method to detect unknown colonies of storm petrel (*Hydrobates pelagicus*). Avocetta 36: 95–96.

[26] Mante A, Debize E (2012) MEDITERRANEAN STORM PETREL Hydrobates pelagicus melitensis, Updated state of knowledge & conservation of the nesting populations of the Mediterranean Small Island. Initiative PIM. 20 p.

[27] Albores-Barajas YV, Riccato F, Fiorin R, Massa B, Torricelli P, et al (2011) Diet and diving behaviour of the Mediterranean storm petrel (*Hydrobates pelagicus melitensis*). Bird Study 58: 208–212.

[28] Cutitta A, Patti B, Basilone G, Bonanno A, Caruana L, et al.. (2006) Fluttuazioni interannuali nell'abbondanza degli stadi larvali di Engraulis encrasicolus e di Sardinella aurita in relazione al riscaldamento delle acque superficiali nello Stretto di Sicilia. 537–540 p.

[29] LaFuente JG, Garcia A, Mazzola S, Quintanilla L, Delgado J, et al (2002) Hydrographic phenomena influencing early life stages of the Sicilian Channel anchovy. Fishery Oceanography 11: 31–44.

[30] Patti B, Bonanno A, Basilone G, Goncharov S, Mazzola S, et al (2004) Interannual fluctuations in acoustic biomass estimates and in landings of small pelagic fish populations in relation to hydrology in the Strait of Sicily. Chemistry and Ecology 20: 365–375.

[31] Sabates A, Martin P, Lloret J, Raya V (2006) Sea warming and fish distribution: the case of the small pelagic fish, Sardinella aurita, in the western Mediterranean. Global Change Biology 12: 2209–2219.

[32] Sanz-Aguilar A, Massa B, Lo Valvo F, Oro D, Minguez E, et al (2009) Contrasting age specific recruitment and survival at different spatial scales: a case study with the European storm petrel. Ecography 32: 1–10.

[33] Lo Valvo F, Massa B (2000) Some aspects of the population structure of storm petrels *Hydrobates pelagicus* breeding on a Mediterranean island. Ringing & Migration 20: 125–128.

[34] Albores-Barajas YV, Massa B, Griffiths K, Soldatini C (2010) Sexual dichromatism in Mediterranean storm petrels *Hydrobates pelagicus melitensis* . Ardeola 57: 333–337.

[35] Grosbois V, Gimenez O, Gaillard JM, Pradel R, Barbraud C, et al (2008) Assessing the impact of climate variation on survival in vertebrate populations. Biological Reviews 83: 357–399. 18715402 10.1111/j.1469-185X.2008.00047.x

[36] North Atlantic Oscillation (NAO). Available: http://www.cpc.ncep.noaa.gov/products/precip/CWlink/pna/nao.shtml. Accessed on 30 July 2012.

[37] Servizio Informativo Agrometeo Siciliano. Available: http://www.sias.regione.sicilia.it/. Accessed on 20 Jul 2012.

[38] Osservatorio delle Acque. Available: http://www.osservatorioacque.it/. Accessed on 30 Jul 2012.

[39] Gremillet D, Lewis S, Drapeau L, van Der Lingen CD, Huggett JA, et al (2008) Spatial match-mismatch in the Benguela upwelling zone: should we expect chlorophyll and sea-surface temperature to predict marine predator distributions? Journal of Applied Ecology 45: 610–621.

[40] Massetti L (2004) Identification and analysis of Mediterranean upwelling areas. Firenze: University of Firenze. 20 p.

[41] Lebreton JD, Nichols JD, Barker RJ, Pradel R, Spendelow JA (2009) Modeling individual animal histories with multistate capture-recapture models. In: Caswell H, editor. Burlington MA: Academic Press. pp. pp. 87–173.

[42] Pradel R (2005) Multievent: An extension of multistate capture-recapture models to uncertain states. Biometrics 61: 442–447. 16011690 10.1111/j.1541-0420.2005.00318.x

[43] Pradel R, Gimenez O, Lebreton JD (2005) Principles and interest of GOF tests for multistate capture-recapture models. Animal Biodiversity and Conservation 28: 189–204.

[44] Choquet R, Lebreton JD, Gimenez O, Reboulet AM, Pradel R (2009) U-CARE: Utilities for performing goodness of fit tests and manipulating CApture-REcapture data. Ecography 32: 1071–1074.

[45] Gauthier G, Milot E, Weimerskirch H (2012) Estimating dispersal, recruitment and survival in a biennially breeding species, the Wandering Albatross. Journal of Ornithology 152: S457–S467.

[46] Choquet R, Rouan L, Pradel R (2009) Program E-SURGE: a software application on fitting multievent models. In: Thompson DL, Cooch EG, Conroy MJ, editors. Modelling demographic processes in marked populations. New York: Springer.

[47] Burnham KP, Anderson DR (2002) Model selection and multimodel inference - a practical information-theoretic approach. New York: Springer-Verlag.

[48] Akaike H (1973) Information theory and an extension of the maximum likelihood principle. In: Petrov BN, Csaki BF, editors; Academiai Kiado: Budapest. pp. 267–281.

[49] Libois E, Gimenez O, Oro D, Minguez E, Pradel R, et al (2012) Nest boxes: A successful management tool for the conservation of an endangered seabird. Biological Conservation 155: 39–43.

[50] Jenouvrier S, Tavecchia G, Thibault JC, Choquet R, Bretagnolle V (2008) Recruitment processes in long-lived species with delayed maturity: estimating key demographic parameters. Oikos 117: 620–628.

[51] IPCC - Intergovernamental Panel on Climate Change. Available: http://www.ipcc.ch/index.htm#.UorD8fn9q9E. Accessed on 18 Nov 2013.

[52] Bogdanova MI, Daunt F, Newell M, Phillips RA, Harris MP, et al (2011) Seasonal interactions in the black-legged kittiwake, Rissa tridactyla: links between breeding performance and winter distribution. Proceedings of the Royal Society B-Biological Sciences 278: 2412–2418. 10.1098/rspb.2010.2601 PMC3125632 21208952

[53] Hemery G, D′Elbée E (1985) Discrimination morphologique des populations atlantique et méditerranéenne de Petrel tempete Hydrobates pelagicus. Oiseaux marins nicheurs du Midi et de la Corse Ann du CROP 2: 63–67.

[54] Cubaynes S, Doherty PF Jr, Schreiber EA, Gimenez O (2011) To breed or not to breed: a seabird's response to extreme climatic events. Biology Letters 7: 303–306. 20943677 10.1098/rsbl.2010.0778 PMC3061172

[55] Renault L, Oguz T, Pascual A, Vizoso G, Tintore J (2012) Surface circulation in the Alboran Sea (western Mediterranean) inferred from remotely sensed data. Journal of Geophysical Research-Oceans 117.

