# Supplementary figures and images for: Climate Driven Life Histories: The Case of the Mediterranean Storm Petrel
Source: PLoS One. 2014 Apr 11;9(4):e94526. doi: 10.1371/journal.pone.0094526 (PMC3984163; doi:10.1371/journal.pone.0094526)

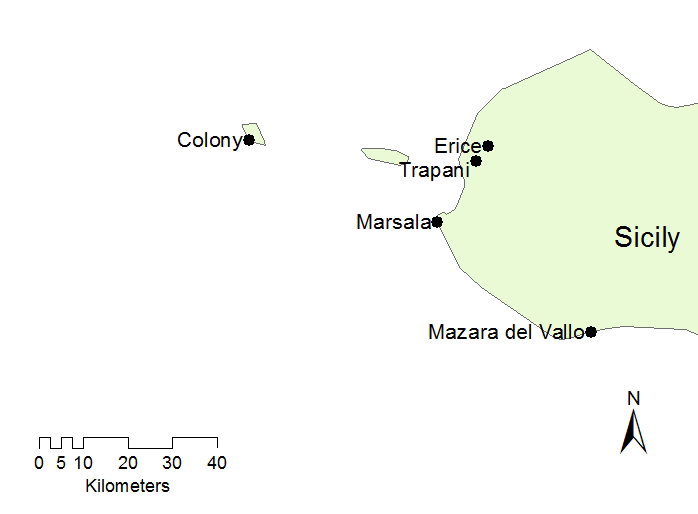

Supplement: Figure S1 — Colony and meteorological stations location. (TIF) [file pone.0094526.s001.tif]

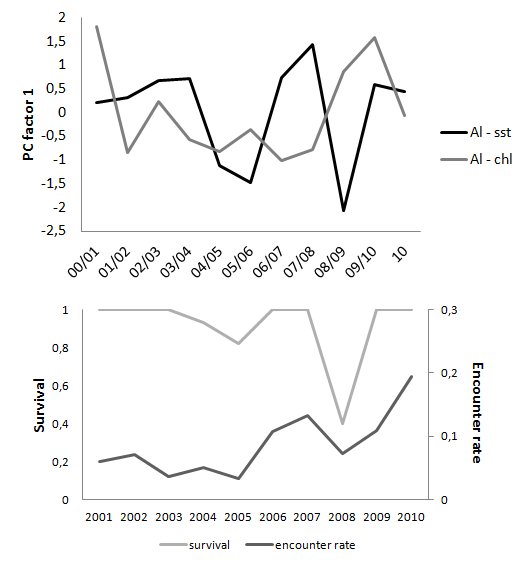

Supplement: Figure S4 — Trends of environmental covariates and demographic traits. a) Time series of sea surface temperature, SST, and chlorophyll concentration, CHL, in the Alboran Sea. b) Estimated survival and encounter rate at Marettimo's colony using Alboran Sea climatic conditions (CI values are nil thus not visible in the graph). (TIF) [file pone.0094526.s004.tif]

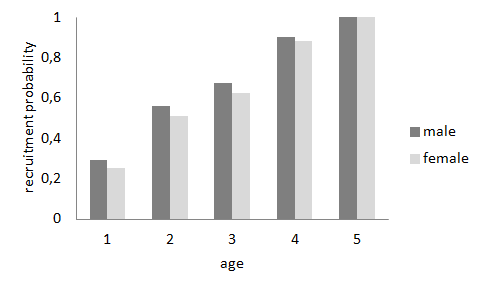

Supplement: Figure S5 — Recruitment probability of males and females storm petrels. (TIF) [file pone.0094526.s005.tif]
