# Supplementary material for: Climate Driven Life Histories: The Case of the Mediterranean Storm Petrel
Source: PLoS One. 2014 Apr 11;9(4):e94526. doi: 10.1371/journal.pone.0094526 (PMC3984163; doi:10.1371/journal.pone.0094526)
Supplement: Material S1 — Detailed descriptions of model implementation in E-Surge. (DOCX) [file pone.0094526.s006.docx]

**Supplementary material 1.** Instructions for implementing the model for survival and recruiting estimates in program E-SURGE.

*Survival and recruitment estimate from encounter history:*

Data are loaded into the program specifying the number of groups (3: males, females and unknown), states (3: “non-breeder“,“breeder“ and “dead“), events (3: “seen as non breeder”, “seen as breeder” and “not seen”), age classes (21) and individual covariates (4: WNAO, WSR, PB and B). Further steps are i) implementation of the matrices structural form in GEPAT interface, ii) setting linear model in GEMACO interface and iii) fixing initial parameters in IVFV interface.

In the GEPAT module in E-SURGE, ‘*’ entries denote the complement of the sum of positive row entries, and ‘-’ entries denote zeroes. For the initial states vector, the transition (decomposed in two matrices, Φ gathering survival probabilities and Ψ gathering probabilities of transition between states) and event matrices introduced above, we have:

$\pi=\left[ \pi* \right]$ $\Pi=\left[ \begin{matrix} * & - \end{matrix} \right],$

$$\Phi=\left[ \begin{matrix} \Phi& - & * \\ - & \Phi& * \\ - & - & * \end{matrix} \right],$$

$\Psi=\left[ \begin{matrix} * & \Psi& - \\ - & * & - \\ - & - & * \end{matrix} \right]$,

$$B=\left[ \begin{matrix} * p & - \\ * - & p \\ * - & - \end{matrix} \right]$$

In the GEMACO interface [[42](#_ENREF_42)], predefined shortcuts are used to specify which parameters are time-constant, time-specific or state-specific (e.g. ‘i’ denotes constancy, ‘t’ means time effect, ‘a’ denotes an age effect, ‘g’ denotes a group effect, ‘x’ denotes a covariate effect, and ‘from’ means that parameters are not equal in each matrix row). For the constant model, we used ‘i’ for initial parameters and ‘a(1,2:21)’ for survival probabilities, ‘a(2,3,4,5,6,7,8,9,10:21)’ for transitions. For the events, we distinguished the first from the following encounter occasions because the encounter history is conditional on being caught in the first period and the following detection probabilities depend on the state and the time occasions. Hence, the formulation for the event is ‘firste+nexte.[from(2).t.a(2:21)+from(1)]’. The model assumes that there are two age classes (young and adult) and there is an age from which the probability to breed ai does not change. We started running a model assuming that there is no change after 10 years. As we obtained that after age 7 there is no improvement in model fitting we reduced the full breeding age to 7 (namely the 6th year of age) thus the syntax for transition was changed to ‘a(2,3,4,5,6,7:21)’. For a full breeding probability we fixed PSI relative to this hypothetical age 1. Thus we tested which is the optimal recruitment age by fixing to 1 the potential age of optimal recruitment, changing years from 1 to 7 in the IVFV step. In the IVFV interface, when specifying initial parameter values, is possible to constrain beta values before running the model.

*Effects of winter climate on recruitment and survival over time:*

Data are loaded into the program specifying the number of groups (2: chicks and adults), states (2: “alive“ and “dead“), events (3: “seen” and “not seen”), age classes (2) and external covariates (2: sst and chl). Further steps are i) implementation of the matrices structural form in GEPAT interface, ii) setting linear model in GEMACO interface and iii) fixing initial parameters in IVFV interface.

In the GEPAT module in E-SURGE we have:

$\pi=\left[ \pi* \right]$ $\Pi=\left[ * \right],$

$$\Phi=\left[ \begin{matrix} \Phi& * \\ - & * \end{matrix} \right],$$

$$B=\left[ \begin{matrix} * p \\ * - \end{matrix} \right]$$

In the GEMACO interface [[42](#_ENREF_42)], we defined 2 shortcuts considering the two groups of input data: group 1= “Juv” individuals ringed as chicks, Juv=[a(1).g(1)], and group 2 = “Ad” individuals ringed as adults, Ad=[a(2).g(1)&g(2)]. For the constant model, we used ‘i’, for initial parameters and for transition we used ‘juv+ad’ for constant model, ‘juv+ad.t’ for time dependent model and ‘juv+ad.[i+t*x(ext cov)]’ for external covariates dependent models. For the events the formulation for the event is ‘firste+nexte.t’.
